# Finnish parliament ASR corpus: Analysis, benchmarks and statistics

**DOI:** 10.1007/s10579-023-09650-7

**Published:** 2023-03-27

**Authors:** Anja Virkkunen, Aku Rouhe, Nhan Phan, Mikko Kurimo

**Affiliations:** Department of Information and Communications Engineering, Aalto University, Espoo, Finland

**Keywords:** Finnish, Speech recognition, Parliament speech data, HMM-DNN, AED, Wav2vec, Metadata

## Abstract

Public sources like parliament meeting recordings and transcripts provide ever-growing material for the training and evaluation of automatic speech recognition (ASR) systems. In this paper, we publish and analyse the Finnish Parliament ASR Corpus, the most extensive publicly available collection of manually transcribed speech data for Finnish with over 3000 h of speech and 449 speakers for which it provides rich demographic metadata. This corpus builds on earlier initial work, and as a result the corpus has a natural split into two training subsets from two periods of time. Similarly, there are two official, corrected test sets covering different times, setting an ASR task with longitudinal distribution-shift characteristics. An official development set is also provided. We developed a complete Kaldi-based data preparation pipeline and ASR recipes for hidden Markov models (HMM), hybrid deep neural networks (HMM-DNN), and attention-based encoder-decoders (AED). For HMM-DNN systems, we provide results with time-delay neural networks (TDNN) as well as state-of-the-art wav2vec 2.0 pretrained acoustic models. We set benchmarks on the official test sets and multiple other recently used test sets. Both temporal corpus subsets are already large, and we observe that beyond their scale, HMM-TDNN ASR performance on the official test sets has reached a plateau. In contrast, other domains and larger wav2vec 2.0 models benefit from added data. The HMM-DNN and AED approaches are compared in a carefully matched equal data setting, with the HMM-DNN system consistently performing better. Finally, the variation of the ASR accuracy is compared between the speaker categories available in the parliament metadata to detect potential biases based on factors such as gender, age, and education.

## Introduction

Automatic speech recognition (ASR) training data, transcribed speech, is expensive to create. There are some publicly funded large-scale efforts to specifically create this type of data, to facilitate language technologies in the chosen language. Commercial agents create private datasets driven by business ventures and internal research projects. Lastly, there are public and private data that have not been explicitly created for ASR research but can be leveraged for that purpose. A common source in many languages has been certain public forums, such as parliaments, which often produce and publish transcripts of their sessions.

This work presents, to the best of our knowledge, the most extensive public monolingual corpus of parliament session data purposed for ASR and the most extensive transcribed public Finnish ASR corpus at a little over 3000 h altogether. We present benchmark results and provide recipe starting points for the data. We leverage and compare two different wav2vec 2.0-based pretrained transformer models, with the best wav2vec 2.0 HMM system yielding the best performance overall. Additionally, we explore how models trained on parliament session speech generalise to other domains and modes of speech. The extent of the data lends itself to comparisons between hidden Markov model (HMM) systems, which are known to already work well at smaller scales and end-to-end models, which are thought to become competitive at larger scales.

New Finnish Parliament session data become available constantly, and our data collection and processing pipeline can be run intermittently, producing new, ever more extensive versions of this corpus. This prompts the question: how much improvement in ASR performance can we expect from more data? Furthermore, how does the data distribution shift over time-do models trained on data from previous electoral cycles perform well on new data, which has new voices and new topics such as the COVID-19 pandemic?

The Finnish Parliament provides a rich set of metadata about the speakers and recordings. These allow us to statistically analyse the dataset and our ASR results on it from multiple viewpoints. In summary, the contributions of this work are:

- Public release of the most extensive transcribed Finnish ASR dataset (3000 h of manually transcribed and automatically segmented and aligned speech), with two temporally distinct subsets and two test sets
- Development of a new pipeline for retrieving and processing Finnish Parliament recordings and transcripts
- Benchmark results, recipe starting points, generalisation study, experiments with large wav2vec 2.0-based models, and an equal data comparison on hidden Markov model systems and end-to-end attention-based encoder-decoder models
- Analysis of the results, as well as the data based on multiple metadata factors

## Related work

**Table 1:** Properties of ASR datasets named in Sect. 2. Lahjoita puhetta and VoxPopuli have both transcribed and untranscribed subsets. Wang et al. (2021) did not define the speaker count for untranscribed VoxPopuli. For Speecon and SpeechDat, we report the size and speaker count of the Finnish subsets. SpeechDat is the only Finnish corpus with telephone-quality speech (8 kHz)

Data set	Size	Speakers	Languages	Domain
Finnish datasets				
DSPCon	10 h	242	fi	Conversational
FinDialogue	10 h	22	fi	Conversational
Finnish parliament	3087 h	449	fi	Political
Lahjoita puhetta	1601 h	17,821	fi	Spontaneous
Lahjoita puhetta (untranscribed)	1597 h	18,825	fi	Spontaneous
Speecon (Finnish subset)	204 h	550	20	Read, spontaneous
SpeechDat (Finnish subset)	236 h	4000	14	Read, telephone
Parliament ASR datasets				
Bern parliament	293 h	224	de	Political
Bulgarian parliament	249 h	572	bg	Political
Croatian parliament	1816 h	310	hr	Political
Czech parliament	444 h	212	cs	Political
Danish parliament	1857 h	434	da	Political
Finnish parliament	3087 h	449	fi	Political
Icelandic parliament	542 h	197	is	Political
Norwegian parliament	140 h	267	no	Political
Valais parliament	40 h	204	de, fr	Political
VoxPopuli	1791 h	4295	16	Political
VoxPopuli (untranscribed)	384,000 h	–	23	Political

Mansikkaniemi et al. (2017) created and studied the first version of the Finnish Parliament ASR Corpus. They extracted 1559 h of ASR data from over 2000 h of video recordings. This data was never publicly released, and the retrieval pipeline used web scraping and obsolete interfaces. The data preparation pipeline was based on AaltoASR.^1^ This preliminary work forms one training subset of our data and contributes the temporally earlier development and test sets. We developed a new retrieval pipeline for the new official open data interface and based our processing pipeline on Kaldi (Povey et al., 2011). We retrieved all data available on the new interface, which forms second, more recent training subset of our corpus. In addition, we pooled all data together to form a combined training subset of 3087 h. We manually corrected and curated a new test set, which covers a later period of time than the first test set. We reported new benchmark HMM-DNN results, and we also reported attention-based encoder-decoder (AED) benchmark results and published HMM-DNN and AED recipes. We applied recent state-of-the-art wav2vec 2.0 models. We analysed the data and modelled errors and biases statistically.

In Table 1, we can see a list of relevant Finnish corpora. Before the Finnish Parliament ASR Corpus, early large-scale efforts to collect Finnish speech data were part of larger international projects to build multi-lingual speech databases-SpeechDat for speech-driven teleservices (Höge et al., 1999) and Speecon for speech-driven interfaces in consumer devices (Iskra et al., 2002). Both databases are around 200 h in size and contain mainly read speech like isolated digits and words, numbers, spellings, dates, commands, and sentences. These databases are publicly available for academic and commercial use, but only for fees ranging from €30,000 to €75,000.

DSPCon (Enarvi, 2018), FinDialogue (Lennes, 2009), and Lahjoita puhetta (Moisio et al., 2022) corpora represent more spontaneous and conversational forms of Finnish speech. DSPCon contains short conversations between students. Similarly, the FinDialogue Corpus is a collection of 10 spontaneous long dialogues between friends. Lahjoita puhetta is a new, considerably larger corpus of spoken and colloquial Finnish made of speech donations collected from the general public. The corpus has both transcribed and untranscribed subsets and covers a large variety of speakers and speaking styles. There are also more databases of spontaneous Finnish, such as Prosovar, but they are currently not publicly available (Lindén et al., 2022).

Besides the Finnish Parliament data, parliament meeting records and transcripts have provided a valuable source of ASR data for many other languages as well. We list examples given here for comparison in Table 1. One of the earliest examples is the MediaParl Corpus for French and German spoken in the Swiss Valais Parliament by Imseng et al. (2012). In recent years, public corpora based on parliament records has also been created for Icelandic (Helgadóttir et al., 2017), Bulgarian (Geneva et al., 2019), Danish (Kirkedal et al., 2020), Czech (Kratochvil et al., 2020), Swiss German (Plüss et al., 2020), Croatian (Ljubešić et al., 2022), and Norwegian (Solberg & Ortiz, 2022). Various event recordings from the European Parliament have also served as raw material for two multi-lingual corpora. First is the large VoxPopuli ASR dataset, which contains both transcribed and untranscribed speech data (Wang et al., 2021). Second is the Europarl-ST dataset, which was primarily created for speech translation needs but can be applied to speech recognition (Iranzo-Sánchez et al., 2020).^2^

## Data preparation

The speech in Finnish Parliament sessions is a mix of planned speech, like opening statements and interpellations, and more spontaneous speech, such as debate. The 200 members of parliament, of whom 94 are women (2022), are elected every four years from 13 districts around Finland. Parliament sessions are held four times a week, and each working year is split into two terms-the spring term from February to June and the autumn term from September to December. Every year roughly 500 h of new video recordings become available.

The data preparation pipeline for the Finnish Parliament speech has been completely redone since the previous iteration described in Mansikkaniemi et al. (2017). The reasons for this are two-fold. First, since the previous iteration, the Finnish Parliament has made changes to their open data interfaces. The first version spanned 2008–2016, and transcripts were crawled directly from HTML pages. Since 2015, the plenary transcripts have been available in a rich XML format with more metadata, such as language labels and member of parliament (MP) ID. In order to use all that metadata, the new dataset spans the years 2015–2020. The second reason is to move to the Kaldi toolkit (Povey et al., 2011), which can be used to implement state-of-the-art models and has a well-tested set of segmentation tools^3^ developed by Manohar et al. (2017). The entire pipeline is available on Github.^4^

### Challenges of the data

The primary challenge of exploiting the plenary sessions as speech recognition data is the length of the plenary recordings. They vary from 15 min to 18 h in length. However, data samples used to train ASR are generally less than 30 s long (Chiu et al., 2019). Computational challenges have limited the length of samples in statistical models in the past (Meyer & Schramm, 2006) and continue to do so in contemporary neural network models (Chiu et al., 2019). Therefore, we need to segment the sessions into smaller pieces more suitable for ASR training.

Mismatches between audio and transcripts form a second challenge because the transcripts are edited for clarity and readability. Furthermore, hesitations, repetitions, and colloquial pronunciations are omitted. Voutilainen (2017) states that transcripts of self-corrections, slips of the tongue, and selected particles are edited for readability in the Finnish Parliament. Additionally, morphological and syntactic features of spoken and spontaneous language are replaced with equivalent written language. In the morphological case, for instance, Voutilainen writes that *me mennään* is changed to *me menemme* (‘we go’).

There are further complications as well. For instance, some speech is left untranscribed, or the speaker is not clearly marked. In our dataset, we wanted to include only speech where the speaker is known, so we needed to be able to skip other speech. The recording may also contain long silent sequences where the camera films the room but microphones are muted if the meeting has started late or there are breaks. Finally, transcripts are not always ordered chronologically, causing a mismatch with the audio. Transcripts always follow the day’s agenda, but in long sessions, the chairperson may first choose to discuss topics that incur less debate.

### Pipeline steps

**Fig. 1:**
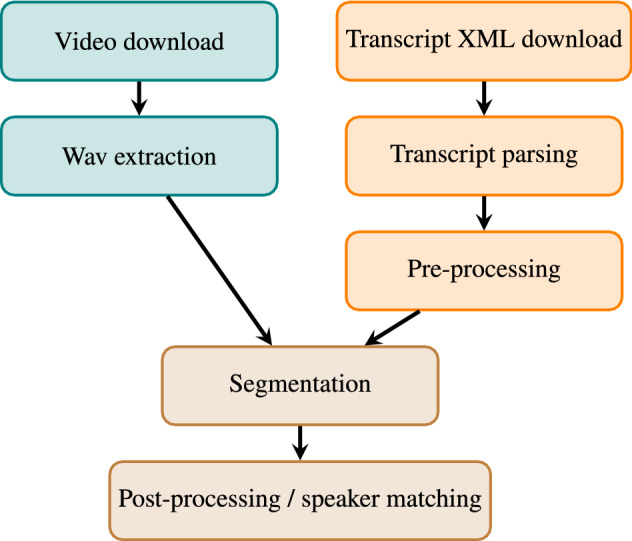
A flowchart showing the steps in the data preparation pipeline

The steps in the data preparation pipeline are visualised in Fig. 1. It begins with downloading the plenary session videos from the video service provider and corresponding XML transcripts from the Parliament’s open data API. After the downloads, a standard 16 kHz single-channel audio wave file is extracted from the video using the FFmpeg tool.^5^ From the XMLs, we parse speech transcripts, speaker name, member of parliament ID, language (Finland is bilingual), political party, title (e.g. chairman, prime minister), and approximate timestamps. Not all speeches have the full metadata, but the speaker’s name and title are always there. Parsed data is saved in a JSON file for human readability and interoperability.

The second step is pre-processing the speech transcripts for the Kaldi segmentation script. For each plenary session, all speech transcripts were pre-processed into one long line of text. The pre-processing maps all Latin characters outside the Finnish alphabet to their closest equivalent in the Finnish alphabet, for example, ø to ö. It also removes transcribed exclamations from other MPs and punctuation, expands digits and abbreviations, and converts all text to lowercase. Since punctuation is removed, samples generated by the segmentation script can start and end in the middle of a sentence. This is in contrast to (Mansikkaniemi et al., 2017), where segmentation was based on sentence boundaries. During pre-processing, we filled in the language labels and speaker IDs in the JSONs if they were missing. Speech was labelled as Finnish, Swedish, or both using predictions from FastText’s language identification model (Joulin et al., 2017). We differentiated the predicted labels from gold standard labels in the JSONs by adding *.p* after the label. Speaker IDs were looked up using the speaker name from a speaker metadata table collected from the Finnish Parliament’s API.

In the third step, audio files and pre-processed texts are segmented with the Kaldi segmentation script. Details of this script are explained in (Manohar et al., 2017). Since speaker turn changes were lost, each session was pre-processed to a single long line of text, and the output of the segmentation script needed to be matched to the JSON transcript for speaker retrieval. For each speech in a plenary transcript JSON, we searched for it in the segmented time-marked conversation file (CTM) generated by the segmentation script. We marked the speaker and statement language in the CTM if the speech was found. After the matching, we kept only segments with one speaker in Finnish.

### Published corpus

With the new pipeline, we processed plenary meetings from 2015 until the end of June 2020, totalling 743 sessions. All in all, the raw audio from these sessions was 2448 h long, of which 1783 h, or approximately 73%, ended up in the final *Train20* training dataset. Out of the raw data, 22% was lost in the segmentation process due to three causes: (1) the audio was silent, e.g. microphone was muted, (2) the speech was not transcribed, or (3) the ASR model could not recognise the speech accurately enough for alignment with the transcript. Of the raw data, 5% was lost in the post-processing step when speaker identities were recovered. We discarded samples that had more than one speaker, as well as samples that contained speech in Swedish.

The size of the previous *Train16* (Mansikkaniemi et al., 2017) training dataset is 1559 h, and it covers sessions from the autumn of 2008 to the summer of 2016. To combine the two datasets, we needed to remove overlapping samples. We decided to drop samples spanning 2015 and 2016 from the old Parliament set so that the *Combined* dataset would cover years 2008–2020. From the new, *Train20* dataset, we removed any samples that overlapped with development or test sets defined in Mansikkaniemi et al. (2017). When combined, we got a training corpus with 3087 h of data and 449 speakers. The full data is available in Kielipankki.^6^

For model development and testing, we used the development and test sets from Mansikkaniemi et al. (2017). In addition, we created a new test set to evaluate domain shifts in the Parliament speeches. The new, *Test20* set is sampled from the segmented plenary meetings of autumn 2020. We listened to and corrected all samples in this set by hand. Details of all the Finnish Parliament data subsets are listed in Table 2.

**Table 2:** Size, speaker count, and abbreviation of the different Finnish parliament ASR corpus subsets

Subset name	Abbreviation	Size	Speakers
Train 2008–2016	Train16	1559 h	357
Train 2015–2020	Train20	1783 h	302
Combined train 2008–2020	Comb	3087 h	449
Development 2008–2016	Dev16	5 h	19
Test 2008–2016	Test16	5 h	21
Test 2020	Test20	5 h	28

**Fig. 2:**
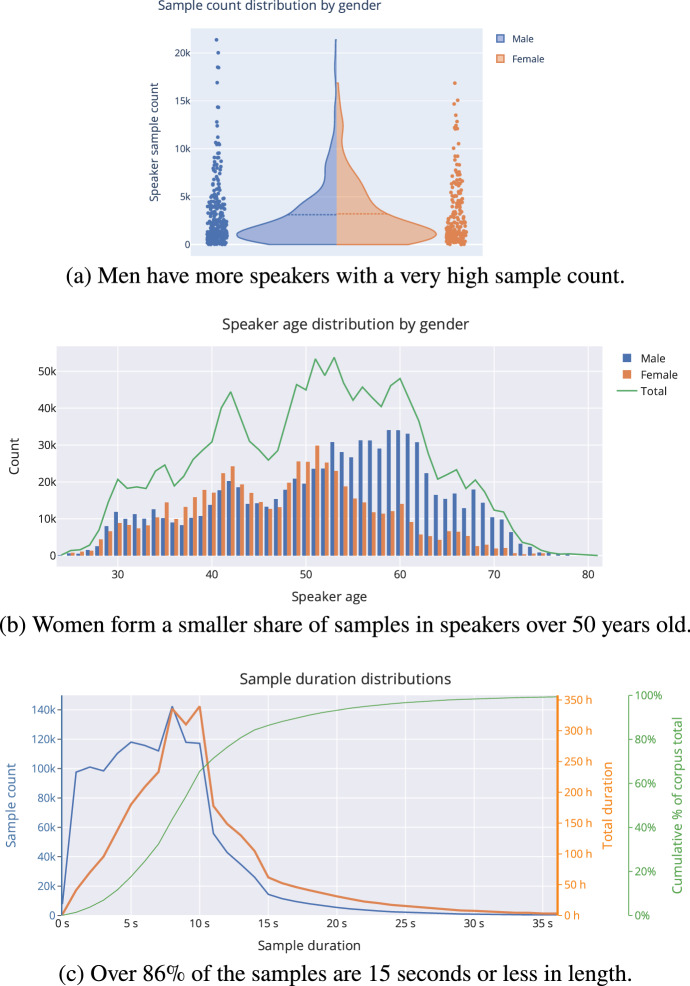
Some characteristics of the *Combined* 2008–2020 training set. In the violin plot at the top, dots correspond to individual sample counts, and the curves display the same data as a density. The dashed line inside the violin plot shows the mean of the distribution

The gender, age and duration statistics of the *Combined* dataset are visualised in Fig. 2. First, Fig. 2a shows how the samples are distributed among speakers and gender. The majority of the speakers have less than 5000 samples each, but there are a few outliers, especially among male speakers. Overall, women account for 40% of both the speakers and the speech audio.

Age-wise, the samples are distributed evenly between genders for speakers under 53 years old, but men dominate among older speakers, as is shown in Fig. 2b. Notably, in most ASR datasets, each speaker gives their sample at a certain age. But in this dataset, samples from a single speaker come from a range of years since MPs usually serve a full electoral term or more.

Different distributions of sample duration are shown in Fig. 2c. Over 85% of the samples are 15 s or shorter in length. This is partially due to the default parameters of the Kaldi segmentation script, which sets the maximum sample length to 15 s. Thus, the newer *Train20* set contains only samples up to 15 s, while the *Train16* set contains longer samples.

## Models

Besides simply demonstrating and evaluating the basic uses of this new data resource, our speech recognition experiments have three main goals. Firstly, these experiments provide benchmarks and recipe starting points for new research. Secondly, we demonstrate how the new *Train20* resource complements the *Train16* set and explore the potential for ASR performance improvements from increasing data sizes in this corpus. Finally, we investigate how models trained on this data generalise to other existing test sets and how different data subsets fare on the more recent and the older test sets.

We start our experiments by optimising Gaussian mixture model (GMM) acoustic models. GMMs are no longer an area of active research. Still, they are typically needed in hidden Markov model-based speech recognition to generate and refine alignments and to cluster the context-dependent phone hidden Markov model (HMM) states. We improved the multi-stage GMM recipe over 27 runs, so that future work on this data can build on a ready, optimised GMM recipe without needing to redo this additional effort. We also provide a benchmark deep neural network (DNN) acoustic model and attention-based encoder-decoder (AED) system. Lastly, we applied state-of-the-art wav2vec 2.0 models in HMM-DNN systems, yielding benchmark results for large pretrained models. Recipes for the experiments are available in Github.^7^

### HMM-GMM

The outline of our GMM recipe is the Kaldi toolkit (Povey et al., 2011) standard. All the GMMs use a tri-state HMM topology with probability density functions (PDF) tied through a phonetic decision tree. As inputs, we used 13 Mel-frequency cepstral coefficients (MFCCs). The recipe begins with a monophone model trained with the shortest 2000 samples. The monophone alignments are then used to train the first $\Delta$+$\Delta \Delta$ triphone model with 100,000 randomly selected utterances. The second triphone model splices together seven feature vectors through a linear discriminant analysis (LDA) transform as its input and adjusts the model through a maximum likelihood linear transform (MLLT). It is trained with 250,000 randomly selected utterances. The third and fourth triphone models add speaker adaptive training (SAT) on top of LDA and MLLT. The former is trained with the same 250,000 samples while the latter is trained on the whole data set.

In addition to the basic Kaldi recipe, we tried other configurations, like leaving out one or two triphone training steps or training from the beginning with a full data set. However, the results did not improve.

The fifth model is the final HMM-GMM used to generate alignments for the neural network training in the next stage. We tuned the number of PDFs (leaves) and the number of Gaussians and updated the model until convergence at 70 iterations. The best result for the *Train20* set used 12,304 leaves in the phonetic decision tree and 501,717 Gaussians-the recipe hyperparameters being 14,000 maximum number of leaves and 500,000 target number of Gaussians.

### Time delay neural networks (TDNN)

Our DNN acoustic model benchmark is based on the Kaldi Librispeech recipe.^8^ To make the recipe simpler and easier to compare against, we did not use i-vectors or speed perturbation. The inputs for this model are 80 Mel-scale filter bank features. The model has 16 factorised time delay neural network (TDNN-F) layers (Povey et al., 2018), an initial LDA splicing layer, and a final feed-forward layer. The model has 18.8 M parameters and was trained for eight epochs, as described by Povey et al. (2016), with the lattice-free maximum mutual information (LF-MMI) criterion and a regularising Cross-Entropy criterion.

We trained a smaller model of 11 TDNN-F layers (7.5 M parameters) and a larger model of 20 TDNN-F layers (26.7 M parameters). Still, in our preliminary experiments, they performed the same or worse. In addition to plain TDNN models, we tested an architecture with three TDNN-F layers and three bidirectional long short-term memory (BLSTM) layers with a total of 46.1 M parameters.

### Language models and lexicon

Because the agglutinative nature of Finnish would require an extensive word lexicon, we opted for a subword-based solution. We developed several n-gram language models (LM) with byte-pair-encoding (BPE) subwords (Sennrich et al., 2016) on both in-domain and out-of-domain data using the VariKN (Siivola et al., 2007) and SentencePiece (Kudo & Richardson, 2018) tools. Neural network language models are left for future work as they are out of scope in this work. All LMs presented here were trained up to 10-grams with a scaling factor of 0.0001 unless otherwise stated. We used a grapheme-based lexicon in this work because Finnish has near phonemic orthography.

For each acoustic model trained on one of the training set splits (*Train16*, *Train20*, and *Comb*), we trained a matching language model with 1750 BPE units using only the transcripts of the given training split. Later, we refer to these LMs simply by the name Transcript LM. We also trained a larger, 19,000 BPE unit, in-domain language model from the 20 M token parliament meeting transcript corpus collected by Mansikkaniemi et al. (2017). This corpus contains complete sentences parsed from the meeting transcripts. It is based on the same plenary meetings data, 2008–2016, as the ASR corpus, so we made a new extended version covering meeting transcripts from 2008 to mid-2020. Using this extended 30 M token corpus, we trained a second in-domain language model with 19,000 BPE units. These two models are later referred to as Parl-20 M and Parl-30 M language models. For all in-domain LMs, we made sure they contained no data from *Test16* or *Test20*.

In addition to in-domain models, we used two out-of-domain language models to evaluate the new acoustic models on out-of-domain test data. We trained a general-domain LM with 19,000 BPE units with the Kielipankki Corpus.^9^ It is a collection of Finnish texts from 1990 s newspapers, journals and books. As this is a large 144 M token dataset, we trained a smaller 5 gram LM for the first recognition pass to create lattices and a 10 gram model for rescoring the lattices. The second model is a word-based, Kneser-Ney smoothed 4-gram for conversational Finnish developed by Enarvi et al. (2017). We use the names General and Conversational LM for these out-of-domain models from here onwards.

### Attention-based encoder-decoder models

Various end-to-end models have recently become mainstream approaches in ASR. We trained end-to-end attention-based encoder-decoder (AED) models (Bahdanau et al., 2016; Chan et al., 2016) to explore this direction. We implemented the models in the SpeechBrain toolkit (Ravanelli et al., 2021). The encoder is a stack of convolutional, long short term memory (LSTM), and feed-forward layers, with four-fold frame subsampling in the convolutional layers. The attention mechanism is a content-and-location aware variant, and the decoder is a stack of gated recurrent units (GRU). The model takes 40-element Mel-scale filter bank log-energy vectors as input and computes a distribution over a vocabulary of 1750 SentencePiece BPE units. The model has 27.7 M parameters. It is trained with dynamic batching, targeting 40 s of audio per batch, for 100 nominal epochs of 10,000 updates, with early stopping. The first 15 nominal epochs use an auxiliary connectionist temporal classification (CTC) loss on the encoder to aid initial convergence (Kim et al., 2017). We tried to improve on this benchmark model in preliminary experiments by building various larger models, but none yielded better results.

We compared the AED models against HMM systems in an *equal data setting* (Rouhe et al., 2021): both paradigms only used transcribed speech as the training data. Since HMM systems typically leverage additional text data and expert lexica, comparing them with end-to-end models only trained on transcribed speech confounds differences in models and learning with differences in the training data. Using just the transcripts for language modelling still follows standard practices, and in Finnish, grapheme lexica are also standard practice due to the transparent orthography. Further care is taken to balance the comparison by using a matching vocabulary with both paradigms and not using augmentation nor i-vectors with either paradigm.

AED models are known to struggle with long-form speech Chiu et al. (2019). When applying the AED models to our out-of-domain Lahjoita puhetta test data, which has long-form recordings, some utterances produce repetitive pathological output, similar to reports by Keung et al. (2020). This is a known issue with AED models on the Lahjoita puhetta test set. To alleviate the problem, we applied a simple post-processing filter where repetitions are allowed to produce a maximum of five tokens, as originally explained by Moisio et al. (2022).

### Leveraging wav2vec 2.0

At the start of the 2020 s, self-supervised learning approaches started to yield remarkably good ASR performance via leveraging vast unlabelled datasets (in the tens to hundreds of thousands of hours) to pretrain very large transformer neural networks (hundreds of millions to billions of parameters). This approach has been spearheaded by the wav2vec family of models, where the wav2vec 2.0 (W2V2) version (Baevski et al., 2020) has provided the best results. The models are pretrained with a contrastive task over quantised, jointly learned representations. After pretraining, the quantised representations are discarded, and the rest of the model is used as an acoustic encoder in ASR tasks, e.g. with CTC, AED, or HMM-DNN approaches (Rouhe et al., 2022; Yi et al., 2021). A key goal of the wav2vec family of models is to enable the building of ASR systems for tasks with very limited data, such as low-resource languages using multi-lingual models and cross-lingual transfer. However, the large pretrained models have also yielded state-of-the-art performance in high-resource tasks, and thus we conducted some experiments with this approach.

We used the standard wav2vec 2.0 Large architecture, which has 317 M parameters and consists of a convolutional front-end followed by a large transformer network. There are many pretrained versions available, of which we compare two: XLS-R,^10^ which is pretrained on 436,000 h of speech covering a wide range of languages and tasks (Babu et al., 2021), and the Uralic V2 model,^11^ which is trained on 42,500 h of Uralic language speech from the VoxPopuli Corpus (Wang et al., 2021). This VoxPopuli subset is also included in the XLS-R training data, being the only source of Finnish data in the XLS-R training data. Finnish belongs to the Uralic language family, and the VoxPopuli Corpus consists of speech from the European Parliament. Thus, although the XLS-R model has seen about ten times more data, the Uralic V2 training corpus is more closely related to the target task. Following the HMM-DNN experiments, we applied this wav2vec 2.0 acoustic model in a Kaldi Chain-like system, with a two-state HMM-topology, left-biphone state-tied tree, and multi-task training with both the LF-MMI and Cross Entropy criteria. Although the Kaldi standard output frame rate is 30 ms, we used 20 ms because that is used by wav2vec 2.0. We started from the HMM-GMM alignments, which are used in state-tying and for Cross-Entropy targets. The models are implemented in SpeechBrain (Ravanelli et al., 2021), and the LF-MMI implementation is from PyChain (Shao et al., 2020). In inference, we used an approach where both the LF-MMI and Cross Entropy head outputs are passed through a LogSoftMax. The resulting two log-likelihoods are linearly interpolated, using the same weighting as in training, and then passed to Kaldi for decoding.

Furthermore, Rouhe et al. (2022) show some promising results by leveraging wav2vec 2.0 on an artificially limited, low-resource-like subset of the *Train16* set.

## Results

In this paper, we present only the main ASR word error rate (WER) results; more can be found on Github.^12^ First, we compare acoustic models (AM) trained with different subsets of training data to study how much the increased training data improves performances. We continue with a similar comparison of language models (LM) trained with different amounts of in-domain text data. Then we evaluate the best TDNN model on various Finnish ASR benchmark test sets. Alongside the HMM-TDNN systems, both on the development data and the test sets, we report results with the wav2vec 2.0-based HMM systems. Finally, we evaluate AED models on the same benchmarks and compare the results to an HMM system trained in an equal data setting, which here means limiting the HMM system to the transcript LM.

### Model development

We started our model development efforts from the HMM-GMM optimisation on the *Train20* set. We used the development set *Dev16* for tuning all the model hyperparameters. The results for the best HMM-GMM are in Table 3.

When we compared *Train20* HMM-GMMs to the *Train16* and *Combined* sets on our development set, we saw that the *Combined* set gives a slight improvement as expected. However, with DNN models, the *Combined* set TDNN-medium performs worse than the same model using *Train20*. Even with the over twice as big TDNN-BLSTM model (see AM parameter counts in Table 4), where we assumed more data would help, the improvement is statistically insignificant.

**Table 3:** *Dev16* WERs of acoustic models trained with different subsets of the training data and evaluated with the Parl-20 M in-domain LM. Results in WER [%]

Acoustic model	Data size (samples)	Train20	Train16	Comb
Monophone GMM	2k	56.24	69.87	61.29
Delta+delta-delta GMM	100k	21.56	21.43	21.34
LDA+MLLT GMM	250k	17.83	17.72	17.63
LDA+MLLT+SAT GMM	250k	16.70	16.77	16.41
LDA+MLLT+SAT GMM	All	14.34	14.42	14.09
TDNN-medium	All	**9**.**98**	10.34	10.28
TDNN-BLSTM	All	10.66	11.06	10.54
With wav2vec 2.0				
W2V2 XLS-R	All + pretraining	9.54	10.17	9.42
W2V2 Uralic V2	All + pretraining	8.73	8.86	**8**.**50**
After clean-up				
LDA+MLLT+SAT GMM	All	14.31	14.22	14.01
TDNN-medium	All	9.37	**8**.**49**	8.69
W2V2 Uralic V2	All + pretraining	7.67	7.47	**7**.**25**

For the *Train20* set, we trained a smaller and a larger TDNN model to see how model size influences the performance of this data. The results are shown in Table 4. It appears that the benchmark TDNN-medium is already a good fit for the *Train20* set. Additionally, we compare the three in-domain language models in the same table. Transcripts make a decent language model, but adding available in-domain data is even better.

We compared the two wav2vec 2.0 models, Uralic V2 and XLS-R, which have the same Large architecture, but different pretraining data. As shown in Table 4, the Uralic V2 model consistently outperforms XLS-R on the *Dev16* development set, even though XLS-R strictly has seen a superset of the data, as explained in Sect. 4.5. For the rest of the wav2vec 2.0 experiments, we focused on the Uralic V2 model.

In both Tables 3 and 4, we observed a saturation effect for the TDNN models. The acoustic model does not improve after increasing the training data from the *Train20* subset to the full *Combined* set. Similarly, the language model does not improve after increasing the training data from the 20 M subset to the full 30 M data. However, the wav2vec 2.0 models do not exhibit this saturation effect: the *Combined* data wav2vec 2.0 models are consistently better than ones trained on *Train16* or *Train20* alone. Perhaps this is due to their large size. Thus wav2vec 2.0 models work well on both small data subsets (Rouhe et al., 2022) and very large sets, as seen here.

In the work of Mansikkaniemi et al. (2017), experiments are performed with data that is cleaned using the Kaldi clean-up tools. To gauge the effect the clean-up has on the training subsets, we ran the clean-up script for each with the final, full data HMM-GMM model as is the Kaldi standard. We trained another LDA+MLLT+SAT HMM-GMM, TDNN-medium model, and wav2vec 2.0 Uralic V2 model with the cleaned data. The clean-up brought minor improvements for the HMM-GMM models, but the improvement is clear for the TDNN-medium and wav2vec 2.0 models. Furthermore, the wav2vec 2.0 models trained on the cleaned *Combined* data yielded the best overall word error rates on the development set.

After clean-up, the *Combined* set demonstrated the same saturation effect with TDNN models as without clean-up. However, when *Train16* and *Train20* are compared against each other, the improvement is clearly more considerable for *Train16*. We attribute this to the different segmentation processes. *Train20* is the product of the Kaldi segmentation script, which uses many of the same functions and steps as the clean-up script. Both only keep data where the speech recognition hypothesis and transcript fully agree, irrespective of sentence boundaries. From *Train20*, the clean-up script removes only 4.9% of the total audio. Meanwhile, *Train16* was segmented at sentence boundaries. This is hard to do with the Finnish Parliament data because, as discussed in Sect. 3.1, the parliament transcribers often make minor edits to the text for the sake of readability. Thus a perfect alignment for many of the sentences is not possible. Consequently, the clean-up script found more alignment errors in *Train16*, leading it to remove 10.3% of the original audio.

**Table 4:** Comparisons of three in-domain language models for uncleaned *Train20* acoustic models on the *Dev16* development set. Language model details are given in section 4.3. Acoustic model parameters are in millions, and results are in WER [%]

Acoustic model	Parameters	Parl-20 M	Parl-30 M	Transcript
Final GMM	0.5 M	14.34	14.38	21.12
TDNN-small	7.5 M	10.24	10.22	15.19
TDNN-medium	18.8 M	9.98	**10**.**02**	**14**.**19**
TDNN-large	26.7 M	**9**.**97**	10.06	14.34
TDNN-BLSTM	46.1 M	10.66	10.58	14.34
W2V2 XLS-R	318.4 M	9.54	9.57	11.04
W2V2 Uralic V2	318.4 M	**8**.**73**	**8**.**69**	**9**.**81**

### Test set results

Next, we focused on the TDNN-medium model and evaluated it on five test sets using the three uncleaned acoustic model training sets and five language models. Results are displayed in Table 5. Two of the test sets, *Test16* and *Test20*, are in-domain, while the remaining three are out of the domain. The Lahjoita puhetta test set contains spontaneous and colloquial speech, Speecon consists of mainly read speech in various conditions, and the YLE test set is made of news and broadcast material. Language models were detailed in Sect. 4.3.

**Table 5:** Comparison of test set results for five different language models (columns) defined in Sect. 4.3 and four different acoustic model types (rows). Three models used the TDNN-medium architecture and were trained on the three training subsets (uncleaned). Additionally, the best uncleaned wav2vec 2.0 model’s (Uralic V2 on the Combined subset) results are presented. Results are grouped by test set, and scores are reported in WER [%]

AM training data	Parl-20 M	Parl-30 M	Transcript	General	Conversational
Test16 set (in-domain)					
TDNN-medium Train20	**6**.**97**	7.05	10.52	10.92	17.83
TDNN-medium Train16	7.83	**7**.**77**	11.17	11.77	18.89
TDNN-medium Combined	**7**.**14**	7.15	9.83	10.60	17.59
W2V2 Uralic V2 Combined	5.98	**5**.**93**	6.54	6.83	13.45
Test20 set (in-domain)					
TDNN-medium Train20	9.83	9.34	**8**.**84**	9.59	17.43
TDNN-medium Train16	14.97	**13**.**17**	13.90	13.89	21.29
TDNN-medium Combined	10.67	9.73	**8**.**76**	10.12	17.57
W2V2 Uralic V2 Combined	6.93	6.78	**6**.**52**	7.09	15.16
Lahjoita puhetta test set (out-of-domain)					
TDNN-medium Train20	66.59	66.20	66.90	64.85	**60**.**05**
TDNN-medium Train16	68.85	67.26	68.41	64.73	**58**.**82**
TDNN-medium Combined	65.48	65.16	64.99	62.79	**57**.**42**
W2V2 Uralic V2 Combined	58.16	57.96	58.47	57.19	**56**.**94**
Speecon test set (out-of-domain)					
TDNN-medium Train20	22.19	21.71	22.12	**14**.**78**	24.54
TDNN-medium Train16	23.73	21.60	22.84	**14**.**33**	24.89
TDNN-medium Combined	22.43	20.42	20.93	**13**.**83**	23.87
W2V2 Uralic V2 Combined	12.42	12.29	12.00	**10**.**61**	18.63
YLE test set (out-of-domain)					
TDNN-medium Train20	25.41	24.89	26.15	**18**.**07**	27.15
TDNN-medium Train16	27.59	26.04	27.58	**17**.**61**	28.37
TDNN-medium Combined	25.49	24.67	24.70	**17**.**04**	26.81
W2V2 Uralic V2 Combined	16.14	15.98	16.21	**13**.**66**	21.31

*Train20* and *Combined* acoustic models perform on the same level for the in-domain test sets, while the *Train16* AM was clearly behind them. The gap is notable, especially on the *Test20* set, which we believe is due to a temporal shift in the data distribution and different segmentation processes. This hypothesis is also supported by the *Train16* AM performing best with the Parl-30 M LM on the *Test20* set because the Parl-30 M LM contains data up to 2020, which the AM is missing.

*Combined* AM performs consistently better on the out-of-domain test sets than the other AMs with all the LMs, except with the Parl-20 M LM. The results for the Lahjoita puhetta test set show how different the colloquial and spontaneous speech is from the formal and planned speech in the Finnish Parliament. The Conversational LM bridges some of that gap, but the WER remains high compared to other test sets. Speecon and YLE test sets get the best results with general-domain LM, and the WERs are lower than for Lahjoita puhetta. This implies that the domains of Speecon and YLE test sets are much closer to Finnish Parliament data than Lahjoita puhetta.

Alongside the TDNN-medium results, Table 5 lists the best wav2vec 2.0 model results. This Uralic V2 wav2vec 2.0 HMM/DNN system trained on the *Combined* data yielded the best results on all test sets. The XLS-R version was also tested here, but the results were slightly ($\approx 10\%$ relative) worse in all cases.

The thorough evaluation of the HMM-DNN systems establishes a robust baseline against which to compare end-to-end models. The HMM-DNN systems and AED models are compared in an equal data setting: besides choices we made in all HMM-DNN experiments, such as grapheme lexicons, this amounts to limiting the HMM-DNN systems to the transcript-based LMs. Table 6 lists the results. The corresponding HMM-DNN system outperforms the AED model in every comparison. The AED results follow the same trends as the HMM-DNN systems, with a Pearson correlation coefficient value of 0.997 across all the listed results.

**Table 6:** Equal data comparison of AED models and HMM systems. Results are in WER [%]. The AED results show the relative WER difference with the corresponding HMM-DNN system in brackets. LP refers to the Lahjoita puhetta test set

	HMM-TDNN-medium			AED		
Test set	Train20	Train16	Comb	Train20	Train16	Comb
Dev16	14.19	13.97	13.08	16.72 (+17.8%)	14.39 (**+3.01%**)	14.09 (+7.72%)
Test16	10.52	11.17	9.83	12.68 (+20.5%)	12.28 (+9.94%)	10.69 (**+8.75%**)
Test20	8.84	13.90	8.76	10.30 (+16.5%)	14.80 (**+6.47%**)	10.15 (+15.9%)
LP	66.90	68.41	64.99	90.06 (+34.6%)	82.52 (**+20.6%**)	79.78 (+22.8%)
Speecon	22.12	22.84	20.93	25.14 (+13.7%)	25.84 (+13.1%)	21.89 (**+4.59%**)
YLE	26.15	27.58	24.70	28.99 (**+10.9%**)	31.19 (+13.1%)	29.37 (+18.9%)

## Analysis and discussion

We start our discussion with the test set evaluations, their implications, and their relation to previous results. Then, we discuss some wav2vec 2.0-specific results separately. Next, we analyse the *Combined* set by decoding it with a TDNN model. From the decoding results, we gather the types of errors made as well as how the errors are distributed through different speaker groups. We continue our discussion with the comparison of the AED and HMM models. Finally, we close our discussion by listing possible future research directions.

### Test set evaluations

As we expected, in-domain models performed the strongest on in-domain test sets. Yet, it is unexpected how the transcript LM gave the best results on the *Test20* set with the *Train20* and *Combined* acoustic models. Since the *Test20* set is a product of the same segmentation script as the *Train20* set, the sentences in the samples can start and end in the middle. On the other hand, the *Train16* set, Parl-20 M LM and Parl-30 M LM are trained on complete sentences. Therefore, we speculate that models trained on complete sentences suffer from a mismatch in the broken sentence structures that appear in the *Test20* set. We think the same phenomenon happens with the general-domain LM that does better than the in-domain Parl-20 M LM on the *Test20* set.

The in-domain results also indicate the Parliament data distribution shifts over time. In Table 5, the *Train16* acoustic model performs 4–5 absolute percentage points worse on the *Test20* set than other AMs when LM is in-domain. Furthermore, on the same test set, the Parl-30 M LM brings improvements for all AMs, and the gains are the biggest with *Train16* AM. We believe this result on Parl-30 M LM indicates that language and topics discussed in the Finnish Parliament change over time. Other causes for the data shift include changes in recording equipment and environment^13^ and different segmentation processes for the two training sets.

The conversational LM is the best match for the spontaneous speech of the Lahjoita puhetta (LP) test set. However, the error rates are still more than twice as large compared to models trained on Lahjoita puhetta data (Moisio et al., 2022). We can identify two significant factors that explain part of the performance gap: (1) differences in vocabulary and (2) LP transcripts, unlike their parliament counterparts, include hesitations, repetitions, and self-corrections. The first one results from Finnish having phonemic orthography. It is possible to reflect even the slightest single-phoneme variations of pronunciation in the transcripts. This leads to a large vocabulary that diverges from the standard Finnish vocabulary.^14^ Enarvi (2018) studies and discusses this phenomenon in more detail. The second factor leaves the Finnish Parliament models unable to predict any disfluencies because the models have learned from the data to ignore all of that.

On the other two out-of-domain test sets, YLE and Speecon, models trained on the Parliament data generalise much better, especially when combined with the general-domain LM. Mansikkaniemi et al. (2017) get lower WER results than us on these test sets. However, they used i-vectors and data clean-up, and the decreases in WER align with the gains expected from the aforementioned methods.

An overall trend in Table 5 shows that both the *Train20* and the *Train16* subsets are sufficient for in-domain tasks. Adding more parliament data did not improve the TDNN models. Conversely, more parliament data was consistently better for out-of-domain tasks, although the gains were small.

Finally, compared to results achieved on the other parliament corpora in Table 1, our models perform in the same 5%-20% WER range. The authors of Croatian (Ljubešić et al., 2022), Czech (Kratochvil et al., 2020), Danish (Kirkedal et al., 2020), and Icelandic (Helgadóttir et al., 2017) Parliament Corpora each trained a TDNN acoustic model using Kaldi and combined it with an in-domain n-gram language model similar to our work. Their test set WER results for this model combination are 16.38%, 7.1%, 14.01%, and 16.38%. It is noteworthy that the training set size^15^ does not directly correlate with performance in this comparison. It is clear that many language-specific factors influence the results despite the shared domain of political speech.

### On the wav2vec 2.0 results

The HMM-DNN systems that leverage wav2vec 2.0 outperform the HMM-TDNN models considerably, showing how well self-supervised pretraining of a very large model on a very large set of untranscribed speech works even at this scale, with thousands of hours of transcribed data. Based on our results, we cannot conclusively determine how much of the wav2vec 2.0 performance is explained by the self-supervised pretraining and how much is due to having a very large model. The pretraining plays a clear role since the two versions used (Uralic V2 and XLS-R) have identical architectures but clearly differing results. The Uralic V2 wav2vec 2.0 model consistently outperforms the XLS-R version on the development data, as seen in Tables 3 and 4. This also extends to all the test sets, both in-domain and out-of-domain, as explained in Sect. 5.2. This is slightly surprising, considering that the XLS-R has seen roughly ten times more data in pretraining and that the XLS-R pretraining data, in fact, even includes the whole Uralic V2 training data as part of the VoxPopuli data. Compared to XLS-R, the Uralic V2 pretraining data covers less variety and is focused on the type of speech that appears in the Finnish Parliament ASR Corpus data. It appears that this leads to a better initialisation for ASR model training on the Finnish Parliament ASR Corpus. Babu et al. (2021) also train even larger versions of the XLS-R model, up to 2 billion parameters. These larger versions probably emphasise the benefits of the increased training data.

The wav2vec 2.0 models don’t mirror the data saturation effect seen with the TDNN-medium model: the wav2vec 2.0 models trained on the full *Combined* data consistently outperform those trained on *Train16* or *Train20* alone. Perhaps the larger capacity of the wav2vec 2.0 transformer architectures allows these models to use increased data, or perhaps there is something inherent in self-supervised pretraining, which is necessary for this.

### Error and bias analysis

To better understand the properties of the Finnish Parliament ASR Corpus, we wanted to study common decoding errors that a simple baseline model makes and how the errors are distributed through speaker groups available in the metadata. Since the 5 hour held-out test set is far too small to analyse the ASR errors properly, we created another setup by taking only 100 h of speech for training and leaving most of the original training data for a huge held-out test set. We made sure there was no overlap in speakers between the two sets. Here we assumed that the models trained with the randomly sampled 100 h would already be good enough to indicate the most challenging speech to recognise. Furthermore, we want to keep as many speakers as possible in the huge held-out test set in order to have a significant representation of the different speaker groups in it.

Specifically, we trained a TDNN-medium model using a dataset to which random speakers’ utterances were added until a total of 40,000 speech samples were reached-this corresponds to roughly 100 h of training data. This 100 h subset ended up having slightly less speech from women and older speakers than the full data. We then decoded the remaining *Combined* set with this TDNN-medium-100 h model and the general-domain LM. We chose the general-domain LM to avoid using the transcripts and other material related to the chosen test data and because a transcript LM of only 100 h would constitute a poor LM.

**Fig. 3:**
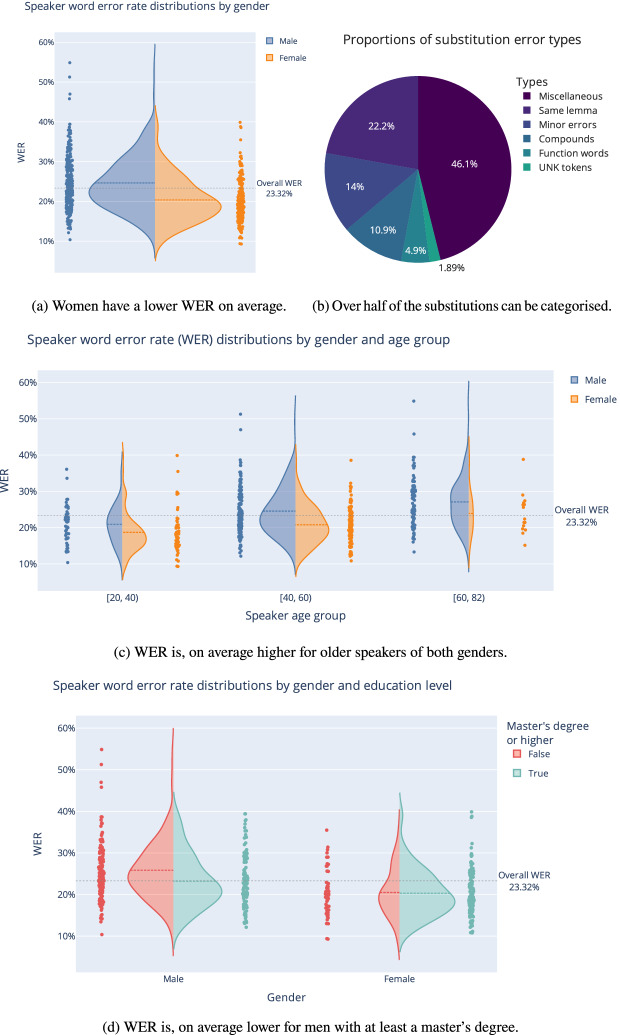
Figures 3**a**, 3**c**, and 3**d** show violin plots of the gender, speaker age, and education level distributions. Figure 3b shows the substitution errors the TDNN-medium-100h model makes. Since a speaker may have contributed samples over many years, we calculate an average age for each speaker from their samples.

Unsurprisingly, the model trained with 100 h is worse than the one trained with over 3,000 h, e.g. 22.64% versus 14.52% on *Dev16*, with the general-domain LM. For the full decoded set that we used in the analysis, the WER is 23.32%. In Figs. 3a, c and d, we visualise how this WER result is distributed among speakers of different gender, age groups and education levels. In the age plot, we have calculated the average age for each speaker from all of their samples because most speakers have contributed samples over several years. Between genders, it is clear that women’s speech is easier to recognise in this corpus despite women’s utterances only making up 22% of the 100 h subset. This difference between genders also persists between the three age groups and education levels. Age-wise, the speech of younger speakers is easier to recognise than that of older ones. However, that observation may be partly explained by the skew toward younger speakers in the 100 h subset. Education level, however, appears to matter only for men. We estimated the effects of speaker dialect (assumed based on the speaker’s birthplace) and the speaker’s political party, but these variables did not have a statistically significant effect on WER in a multivariate ordinary least squares model.^16^

In addition to the speaker distributions, we studied the types of substitution errors the TDNN-medium-100 h model makes. We were able to categorise over half of the substitution errors into recognisable types, as shown in Fig. 3b. In 22.2% of the errors, the reference word and ASR hypothesis have the same lemma. For an agglutinative language like Finnish, it is reasonable that there are many errors related to affixes and inflexions. Minor errors appear in 14% of the cases. We consider substitution error as minor when reference and hypothesis words have different lemmas and edit distance is, at maximum, either one for words up to four characters or two for longer words. Of the errors, 10.9% are related to compound words; separate words are incorrectly compounded or compounded words are separated in the hypothesis. Compounding mistakes significantly affect WER because they imply another insertion or deletion error as well. Function word errors, 4.9 %, occur when one function word is substituted with another. After listening to a few samples, we think many of these are related to the clarity and readability edits parliament transcribers make in the transcripts. The few UNK errors are caused by the UNK tokens the Kaldi segmentation script adds to the segment transcripts to mark unknown acoustics.

### AED versus HMM

End-to-end AED models can be trained on the *Train20*, *Train16*, and the *Combined* datasets without any pre-processing steps. We took care to create a balanced comparison between the AED models and HMM systems: in Table 6, both models used the same data (just transcribed speech), neither model leveraged augmentation/i-vectors, and the total learned parameter counts were about the same (27.7 M for AED vs 18.8 M in HMM acoustic model + 10 M parameters in the n-gram LM). The AED models consistently trailed the corresponding HMM systems in WER. Although in absolute numbers, the AED models fare worse; relatively, they seem to generalise as well as the HMM systems, except for the Lahjoita puhetta data, where the AED models essentially failed.

The AED models and the HMM systems with transcript LMs improve on the *Combined* data, compared to the *Train20* or *Train16* sets alone. We hypothesise that these improvements result mainly from seeing a larger amount of text because the HMM-TDNN system results in Table 4 suggest that for in-domain acoustic modelling, the *Train16* and *Train20* datasets can already be sufficient for some models.

### Future work

The HMM system recipe provides a tuned HMM-GMM system, which can serve to speed up neural network acoustic model research. Furthermore, the full recipe serves as a benchmark. Future work in neural language model rescoring could improve both in-domain and out-of-domain results.

Our recipe for AEDs provides a starting point and benchmark for future research, where Transformer architectures and other larger models could improve the results and even close the performance gap with HMM systems. Additionally, shallow fusion neural language models trained on the 30 M token corpus would be a worthwhile experiment, although the resulting system is no longer trained end-to-end.

This corpus has a relatively rich set of metadata, making it suitable for many speech classification/diarisation tasks, such as speaker recognition. A rare possibility is a longitudinal study of how the representatives’ speech changes over time. The visual stream in the parliament video recordings provides a way to study audio-visual and multimodal speech recognition of Finnish. Public meeting data can be collected from other sources to enrich the corpus further. For instance, some city and local councils in Finland record their meetings and make them available online.^17^ Even if there would be no meeting transcripts available, the data could be harnessed for unsupervised training.

## Conclusion

In this paper, we presented a new, extended version of the Finnish parliament ASR Corpus. With over 3000 h of data, it is the largest transcribed Finnish ASR Corpus we know. The corpus has two official temporally distinct subsets, two temporally distinct, manually corrected test sets, and a development set. We developed benchmark models for three ASR approaches-HMM-GMM, HMM-DNN, and AED.

Our optimised HMM-GMM recipe can be leveraged to kick-start new research. The HMM-DNN and AED recipes provide starting points and benchmarks. Leveraging wav2vec 2.0-based large transformer models is beneficial even with 3000 h of transcribed speech, and our comparison can help to choose an appropriate pretrained version. Despite the large scale of the data, the HMM-DNN approach consistently outperformed the AED approach when compared in a matched equal data setting in our experiments. The experiments show that this dataset is suitable for training ASR systems for many types of planned or formal Finnish, but our models do not generalise to colloquial speech.

The rich metadata allowed us to analyse the errors our models make. Women, younger representatives, and those with at least a master’s level education have lower word error rates than their counterparts.

New parliament sessions can be processed with our new retrieval and segmentation pipeline as it becomes available. Our results suggest that on the temporally older test set performance has already reached a plateau at the current scale for HMM-TDNN models. However, models trained on the older training subset do not perform as well on the newer test set, suggesting that new data is necessary to keep up with some shifting data characteristics. Finally, larger wav2vec 2.0 models can leverage the added data, and thus continued data retrieval may yield better state-of-the-art model performance.

## Footnotes

1 https://github.com/aalto-speech/AaltoASR.

2 We did not find the total hours or speaker count for the Europarl-ST dataset, so we omitted it from Table 1.

3 https://github.com/kaldi-asr/kaldi/blob/master/egs/wsj/s5/steps/cleanup.

4 https://github.com/aalto-speech/fi-parliament-tools.

5 https://ffmpeg.org.

6 http://urn.fi/urn:nbn:fi:lb-2021081105.

7 https://github.com/aalto-speech/fin-parl-models.

8 https://github.com/kaldi-asr/kaldi/blob/66f5434/egs/librispeech/s5/local/chain/tuning/run_tdnn_1d.sh.

9 http://urn.fi/urn:nbn:fi:lb-201403268.

10 https://huggingface.co/facebook/wav2vec2-xls-r-300m.

11 https://huggingface.co/facebook/wav2vec2-large-uralic-voxpopuli-v2.

12 https://github.com/aalto-speech/fin-parl-models.

13 The Finnish Parliament building was under renovation from 2015 to 2017. Plenary sessions were held in a different room during that time.

14 Of the 580,710 words in the *Combined* set, only 95,367 or 16.4% are in common with the 424,605 words of Lahjoita puhetta Corpus.

15 Ljubešić et al. (2022) used only 66 h out of the 1,816 h in their corpus to train the TDNN model.

16 In the model, the response variable is the speaker’s mean WER computed from the utterance WERs. We have 433 speakers in total. The explanatory variables consist of five categorical variables (number of categories in parenthesis): gender (2), age group (3), has master’s degree (2), political party (12), and dialect group (5). For gender, age group and education, the p-value is $p < 0.05$, but not for the rest. $R^2$ for the model is 0.232.

17 See for example (only available in Finnish/Swedish) https://www.raasepori.fi/etusivu/paatoksenteko/esityslistat-ja-poytakirjat/.
